# Diagnostic accuracy of intraoperative brainstem auditory evoked potential for predicting hearing loss after vestibular schwannoma surgery

**DOI:** 10.3389/fneur.2022.1018324

**Published:** 2022-12-15

**Authors:** Feng Gu, Xingyu Yang, Zilan Wang, Xin Tan, Tao Xue, Zhouqing Chen, Zhong Wang, Gang Chen

**Affiliations:** ^1^Department of Neurosurgery and Brain and Nerve Research Laboratory, The First Affiliated Hospital of Soochow University, Suzhou, Jiangsu, China; ^2^Department of Neurology, The Affiliated Suzhou Hospital of Nanjing Medical University, Suzhou Municipal Hospital, Suzhou, Jiangsu, China; ^3^Department of Neurosurgery, Beijing Tiantan Hospital, Capital Medical University, Beijing, China

**Keywords:** brainstem auditory evoked potential (BAEP), vestibular schwannoma, diagnostic accuracy test, meta–analysis, intraoperative neuromonitoring

## Abstract

**Objective:**

This meta-analysis evaluated the diagnostic value of intraoperative brainstem auditory evoked potential (BAEP) for predicting post-operative hearing loss.

**Methods:**

Research articles in MEDLINE, Embase, and Cochrane Library databases were searched and selected up to 20 January 2022, and data were extracted following a standard procedure. A diagnostic accuracy test meta-analysis was performed using a mixed-effect binary regression model.

**Results:**

A total of 693 patients from 15 studies were extracted. The change in intraoperative BAEP showed high sensitivity (0.95) but low specificity (0.37), with an area under the curve of 0.83. Diagnostic accuracy of the loss of potentials showed high sensitivity (0.82) and specificity (0.79). The area under the curve was 0.88. No factor was found to account for the heterogeneity of the results according to the meta-regression and subgroup analyses (all *P*-values > 0.05).

**Conclusions:**

Our results showed that the loss of BAEP has meaningful value for predicting hearing loss after vestibular schwannoma surgery. The change in BAEP is also important for its high sensitivity during hearing preservation surgery.

## Introduction

Vestibular schwannoma (VS), also known as acoustic neuroma, is the most common neoplasm of the cerebellopontine angle. Its common complications, including hearing loss, vertigo, and tinnitus, severely affect patients' quality of life (1). The major and specific clinical symptom of VS is progressive sensorineural hearing loss, which occurs in

up to 90% of patients (2). Complete tumor removal with the preservation of neurological functions can be achieved with surgery. Alternative treatment methods include watchful waiting and stereotactic radiation (3). However, patients with a preserved hearing before surgery are at risk of suffering from post-operative hearing loss if the surgery aimed at complete tumor removal is chosen. Therefore, it is always a trade-off between the extent of tumor removal and cranial nerve protection.

Recently, intraoperative neuromonitoring has been frequently used to assess nerve function during VS resection (4). The use of facial nerve monitoring has been reported to better preserve post-operative facial nerve function (5). During surgery aimed at preserving hearing in patients with residual or normal hearing, the most commonly applied technique is brainstem auditory evoked potential (BAEP) (5) which is sensitive to the conduction velocity change (6). A meta-analysis confirmed that BAEP has remarkable value in the VS diagnosis (7) and can also influence the extent of resection during VS operations to avoid hearing loss caused by microsurgery (4, 6).

Brainstem auditory evoked potential is also used to predict post-operative hearing loss. A meta-analysis by Thirumala et al. showed that the loss of intraoperative BAEP responses had high specificity for predicting hearing loss in the microvascular decompression (8). However, the prognostic value of BAEP for post-operative hearing loss in the resection of vestibular schwannoma remains unclear with a lack of high-level evidence-based medicine proof. Whether intraoperative BAEP can predict post-operative and long-term hearing function has yet to be solved. Hence, we conducted a diagnostic test accuracy meta-analysis to investigate the sensitivity and specificity of BAEP for predicting post-operative hearing loss of patients with VS.

## Methods

### Methods and materials

This diagnostic test accuracy meta-analysis was conducted according to the Preferred Reporting Items for Systematic reviews and Meta-Analyses (PRISMA) statement (9).

### Literature search

A systematic search of MEDLINE, Embase, and Cochrane Library databases was conducted for any research possibly eligible for this meta-analysis. The final search was on 20 August 2021. Both Medical Subject Headings (MeSH) and free-text search terms were used. We also searched the reference lists of studies included for potential additional research. The search strategy details are shown in Supplementary Digital Content 1.

### Selection criteria

Eligible research was selected for the meta-analysis if all the following criteria were met: (1) more than 10 participants were included; (2) all the participants were diagnosed with histologically confirmed VS; (3) hearing of all the participants was present before surgery; (4) hearing outcome was assessed with an objective value including pure tone average and speech discrimination score; (5) details of the intraoperative BAEP results and hearing outcomes were reported, allowing the calculation of diagnostic accuracy metrics; (6) written in English; and (7) human research. The surgical approach (i.e., retrosigmoid) was not considered a selection restriction. The selection procedure was accomplished by two individual authors.

### Data extraction

Research data were extracted independently by two of the authors and checked by a third author. Microsoft Office Excel 16.0 was used to enter and analyze data including study designs; the number of patients; mean age and tumor size (if available); gold standard; true positive, false positive, false negative, and true negative events; and follow-up period.

To investigate the influence of different intraoperative BAEP results on diagnostic value, two analysis groups were formulated according to two definitions of positive events. The positive event definition for the intraoperative BAEP change was that the patient's intraoperative BAEP decreased in amplitude or increased in latency during or at the end of the operation. The threshold change was defined by the original study authors. The positive event definition for intraoperative BAEP loss was that the patient's intraoperative BAEP was lost during or at the end of the operation. The classification was completed individually by two of the authors. The first author independently extracted the data, which were then checked by the second author; a senior author resolved conflicts.

### Quality assessment

The revised tool for the quality assessment of diagnostic accuracy studies (QUADAS-2) was applied for judging the quality of the included articles (10). This procedure was accomplished individually by two of the authors, and disagreements were resolved by a senior author. Review Manager 5.3 (Nordic Cochrane Center, The Cochrane Collaboration, Copenhagen, Denmark) was used for quality assessment.

### Statistical analysis

Sensitivity, specificity, and positive and negative likelihood ratios (LRs) were calculated for each study along with a 95% confidence interval (CI). A bivariate mixed-effects binary regression model was applied. The separate and overall sensitivity and specificity results are also presented as forest plots. The heterogeneity of the meta-analysis was evaluated by Cochran's *Q*-test and *I*^2^ index (11). To find potential heterogeneity, we performed meta-regression and subgroup analyses according to the study design, follow-up period, sample size, publication time, and tumor size. We applied the hierarchical summary receiver operating characteristic (HSROC) model with the credible and prediction regions to pool the overall sensitivity and specificity results (12). Fagan's plot was calculated from the cumulative data to determine the clinical utility of BAEP and pre-test probability, depending on the incidence of post-operative hearing loss. LRs were presented by likelihood ratio scattergram. Positive LR >10 and negative LR < 0.1 were expected to indicate meaningful changes in post-test probability. Deeks' funnel plot was also applied to assess potential publication bias (13). STATA software 16.0 (STATA Corp., College Station, Texas, USA) was used for statistical analyses.

## Results

### Search and selection results

**Figure 1:**
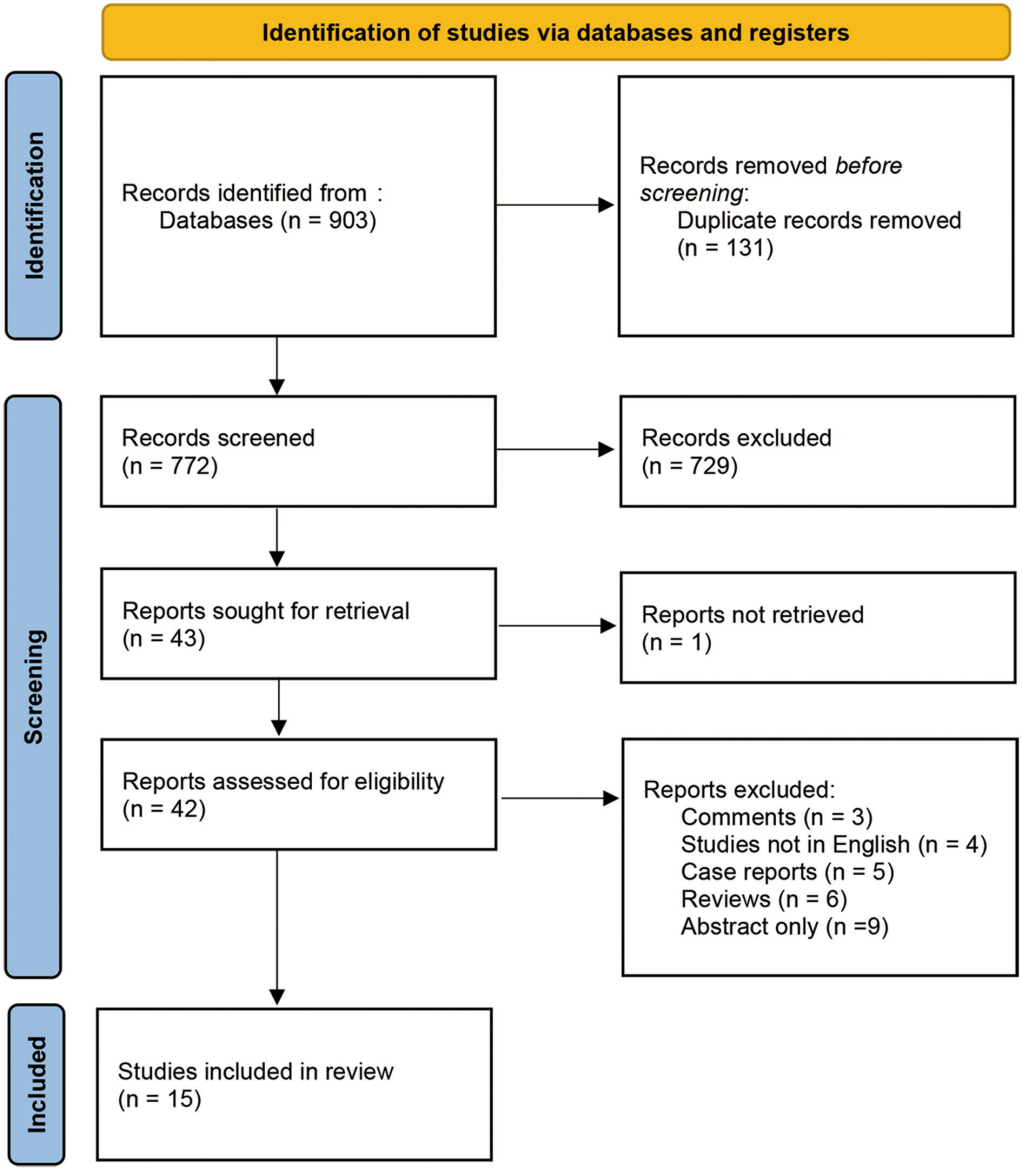
PRISMA meta-analysis search strategy flow chart.

We identified 903 records from various databases up to 20 August 2021. After removing duplicates, 772 records were screened by title and abstract. Among these, 729 records were excluded as they were not directly relevant to the study purpose. The remaining 43 records were assessed with full-text articles for eligibility. The final 15 studies fulfilled the selection criteria and were included in analyses. The procedure details are shown in Figure 1. All 15 studies included an intraoperative BAEP change group, and 13 studies included an intraoperative BAEP loss group.

### Patient characteristics

A total of 693 patients with VS from 15 studies were included in the meta-analysis. Among the included studies, three were prospective and 12 were retrospective. Among the cumulative patients, 359 patients experienced post-operative hearing loss (51.95% incidence). Sample sizes ranged from 11 to 126. Detailed patient characteristics are shown in Table 1.

**Table 1:** Baseline characteristics of included studies.

**Study**	**Country**	**Study design**	**Cases**	**Mean age (years)**	**Mean tumor size (mm)**	**Threshold of change**	**Standard of hearing loss**	**Number of hearing loss events**	**Follow-up period**
Ojemenn et al. (14)	US	Prospective	16	42.8	19.5	Any change	SDS	7	Post-operation
Abramson et al. (15)	US	Retrospective	16	48.2	39.0	BAEP loss	SDS	12	Mean 17.2 months
Strauss et al. (16)	Germany	Prospective	11	50.5	28.6	Gradual loss of one of the wave I–V	PTA	7	3 months
Harper et al. (17)	US	Retrospective	91	45.0	23.0	>50% amplitude and >1 ms latency of wave V	PTA and SDS	60	3 months
Dornhoffer et al. (18)	Germany	Retrospective	72	49.9	–	Higher than or equal to 6.8 ms increase in Wave V latency	PTA and SDS	31	Post-operation
Colletti et al. (19)	Italy	Retrospective	18	49.0	12.1	Higher than 0.5 ms increase in wave V latency	PTA and SDS	6	Post-operation
Neu et al. (20)	Germany	Retrospective	70	–	28.0	Discontinuous identification of waves I and/or V	PTA	43	1 year
Schmerber et al. (21)	France	Retrospective	14	49.0	18.0	Latency shift exceeds 0.5 ms	PTA and SDS	7	1 year
Bischoff et al. (22)	Germany	Retrospective	92	53.0	22.6	Discontinuous identification of waves I and/or V	PTA and SDS	58	1 year
Yamakami et al. (23)	Japan	Retrospective	22	51.0	-	Interaural latency of wave V > 0.2 ms	PTA	2	48 months
Phillips et al. (24)	US	Retrospective	40	47.0	5.2	Wave V delayed or (and) attenuated	PTA and SDS	17	2 weeks
Aihara et al. (25)	Japan	Retrospective	36	48.4	–	Interaural difference of Wave V > 1.12 ms	PTA and SDS	22^*^	2 weeks
Hummel et al. (26)	Germany	Prospective	44	47	–	BAEP class deterioration	PTA and SDS	27	7–10 days
Mastronardi et al. (27)	Italy	Retrospective	25	44.3	20.4	Variable morphological alteration and latencies	PTA and SDS	12	4 months
Sun et al. (28)	US	Retrospective	126	48.6	9.9	ABR absent	PTA and SDS	48	22 months

SDS, speech discrimination score; PTA, pure tone average; –, not reported;

* 22 patients suffered hearing loss classified as GR class III.

### Study quality and risk of bias

**Figure 2:**
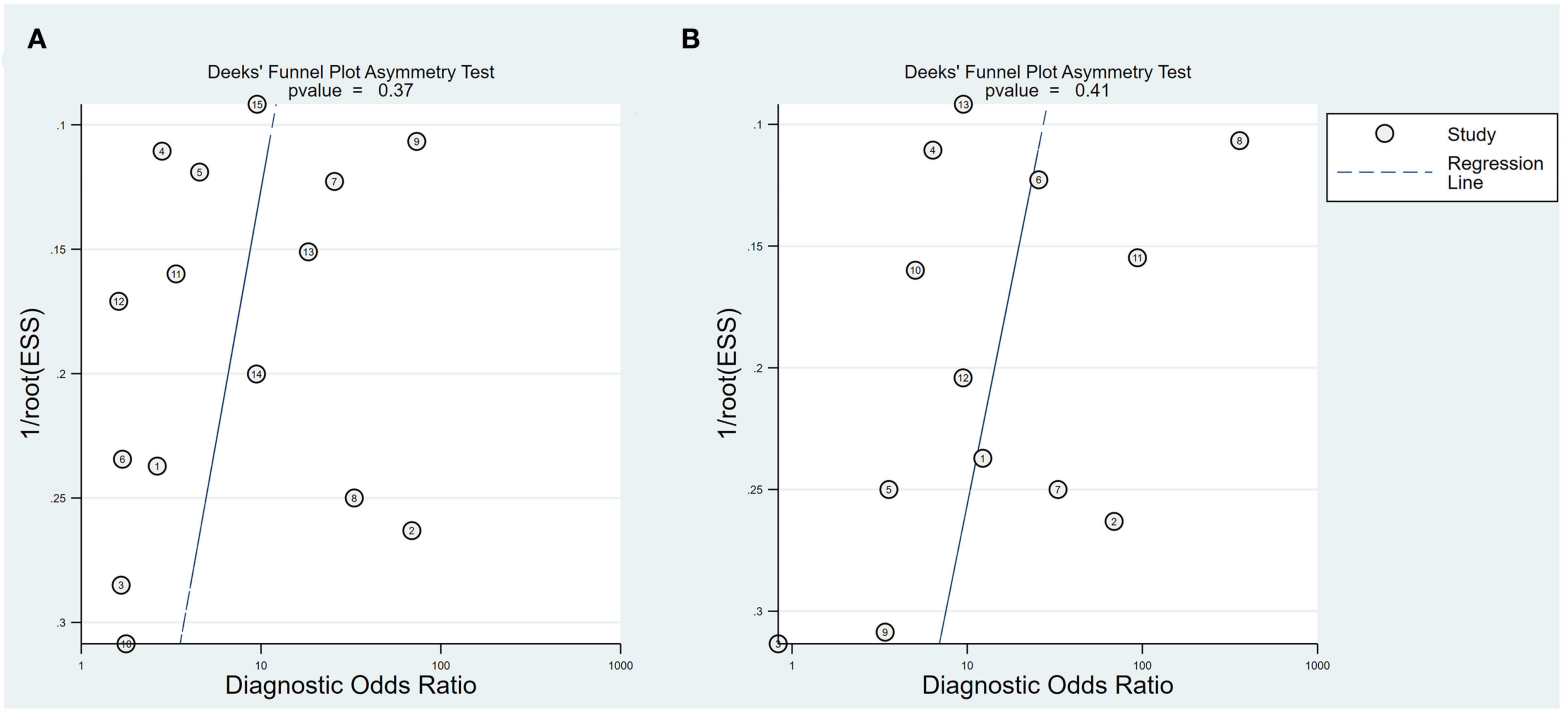
Deeks' funnel plot. **(A)** Deeks' funnel plot of intraoperative BAEP change group; **(B)** Deeks' funnel plot of intraoperative BAEP loss group.

Study quality assessments based on the QUADAS-2 criteria are shown in Supplementary Digital Contents 2, 3. Seven studies did not present an obvious risk of bias or applicability concerns on patient selection, index test reference standard, or flow and timing. However, three of the 15 studies only reported post-operative hearing and two reported post-operative hearing after no more than two post-operative weeks, contributing to potential follow-up bias. Deeks' funnel plot asymmetry test showed no publication bias in either the intraoperative BAEP change group (*P*-value = 0.37) or the intraoperative BAEP loss group (*P*-value = 0.41) (see Figure 2).

### Diagnostic accuracy

#### Intraoperative BAEP change

**Figure 3:**
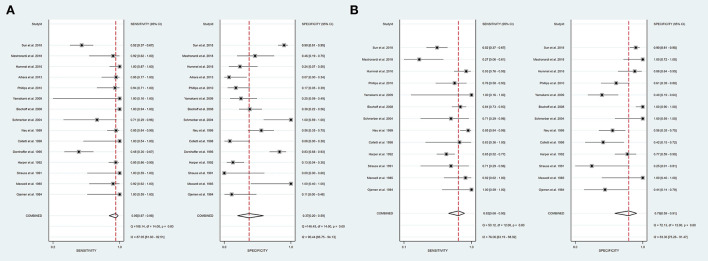
A forest plot of overall sensitivity and specificity. **(A)** A forest plot of intraoperative BAEP change group; **(B)** A forest plot of intraoperative BAEP loss group.

**Figure 4:**
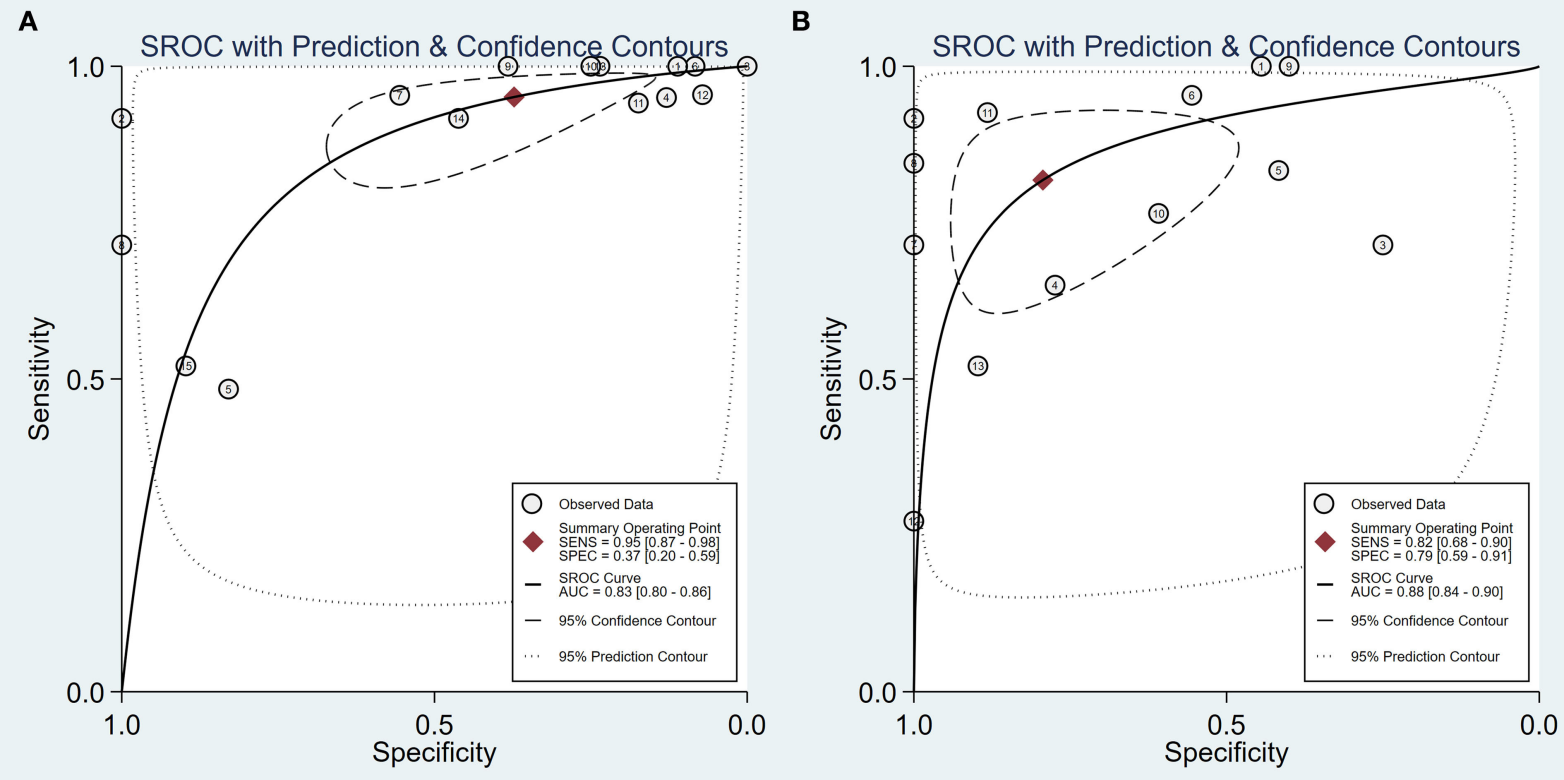
The hierarchical summary receiver operating characteristic curve. **(A)** An HSROC curve of intraoperative BAEP changed group; **(B)** HSROC curve of intraoperative BAEP lost group.

**Figure 5:**
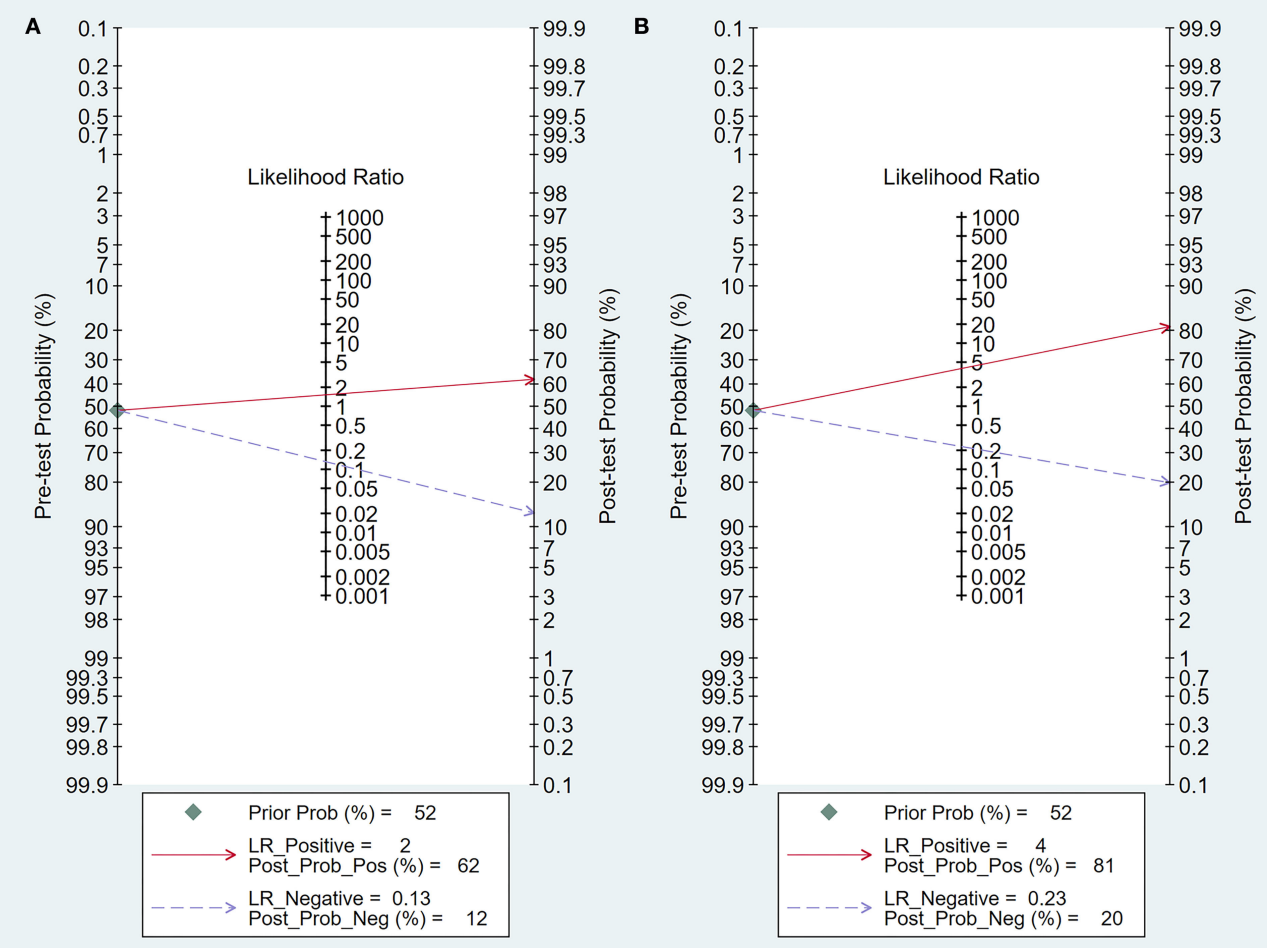
Fagan's plot of post-test probability. **(A)** Fagan's plot of intraoperative BAEP change group; **(B)** Fagan's plot of intraoperative BAEP loss group.

Intraoperative BAEP change criteria differed among the studies and are shown in Table 1. Examining intraoperative BAEP change for its post-operative hearing loss diagnostic value, the overall sensitivity was 0.95 (95% CI: 0.87–0.98) and the overall specificity was 0.37 (95% CI: 0.23 to 0.59). However, the heterogeneity of both sensitivity and specificity was relatively high. The Q-value was 108.14 for sensitivity and 146.45 for specificity, and *I*^2^ was 87.05% for sensitivity and 90.44% for specificity. Detailed sensitivity and specificity scores for each study are shown in Figure 3A. The HSROC curve is shown in Figure 4A, with an area under the curve (AUC) of 0.83. The pre-test probability was 51.9%, and the Fagan plot showed that, with the inclusion of changed intraoperative BAEP, positive post-test probabilities reached 62% and negative post-test probabilities reached 12% (see Figure 5A). The likelihood ratio scattergram showed that the pooled positive LR was < 10 and the pooled negative LR was > 0.1 (see Supplementary Digital Content 4).

#### Intraoperative BAEP loss

The diagnostic value of intraoperative BAEP loss for post-operative hearing loss showed a sensitivity of 0.82 (95% CI: 0.68 to 0.90) and a specificity of 0.89 (95% CI: 0.59–0.91). The heterogeneity of sensitivity and specificity was relatively lower compared with the BAEP change group. The Q-value was 50.12 for sensitivity and 76.06 for specificity; *I*^2^ was 72.13% for sensitivity and 83.36% for specificity. Detailed sensitivity and specificity for the separate studies are shown in Figure 3B. The HSROC curve is shown in Figure 4B, and the AUC was 0.88. As shown in Figure 5B, the Fagan plot demonstrates that, with the addition of changed intraoperative BAEP, positive post-test probabilities reached 81%, and negative post-test probabilities were 20% when the pre-test probability was 51.9%. The likelihood ratio scattergram demonstrates that the pooled positive LR was < 10 and the pooled negative LR was > 0.1 (see Supplementary Digital Content 5).

### Heterogeneity sources

Among the factors analyzed, none was found to affect the heterogeneity of both the intraoperative BAEP change group and the intraoperative BAEP loss group (all *P*-values >0.05) (see Supplementary Digital Contents 6, 7). Detailed overall subgroup sensitivity and specificity are presented in Supplementary Digital Contents 8, 9. The subgroup analysis of tumor size only included studies reporting tumor size, and no effect was found in either outcome group (see Supplementary Digital Contents 10, 11).

## Discussion

In this study, we calculated diagnostic accuracy using a meta-analysis focused on the sensitivity and specificity of intraoperative BAEP to predict hearing loss after VS resection among the cumulative 693 patients included in 15 studies (14–28). Our results demonstrate that intraoperative BAEP has a meaningful diagnostic function for post-operative hearing loss. If the intraoperative BAEP change is used to predict hearing loss after VS resection, sensitivity reaches 0.95 and the negative LR is 0.13. High sensitivity and negative LR indicate an exclusion effect, with a low false negative rate from a change in BAEP for predicting post-operative hearing loss. The high sensitivity could provide a warning value for monitoring hearing function during VS surgery, allowing the neurosurgeon to decide whether to proceed with tumor resection or to stop. High sensitivity is meaningful among patients with bilateral VS for the potential protection of unilateral hearing. However, the specificity is only 0.37, indicating a relatively high rate of false positive events.

We also evaluated the loss of intraoperative BAEP as another factor in predicting hearing loss. Analyses showed that the overall sensitivity was 0.82 and specificity was 0.79, with an AUC of 0.88, representing a higher accuracy (29). This result revealed a notable, meaningful prognostic value of intraoperative BAEP for predicting hearing loss.

However, the analysis of heterogeneity was relatively significant for both the intraoperative change group and the loss group. To identify the source of heterogeneity, meta-regression and subgroup analyses were conducted with the study design, follow-up period, sample size, publication time, and tumor size. None of these factors significantly affected the heterogeneity. Like most intraoperative neuromonitoring technologies, BAEP lacks a diagnostic standard. Thus, alert criterion and monitoring efficacy vary across institutions. Likewise, in our analysis, the meaning of change, including that of amplitude or latency, was also according to the original authors' definitions, possibly contributing to heterogeneity.

As a far-field potential, BAEP requires signal averaging interval. This causes a delay between actual surgical trauma and the observed waveform change. Near-field monitoring techniques such as cochlear nerve compound action potential and electrocochleography might provide more sensitive signals. However, electrocochleography would not reflect the injury on the intracranial portion of the eighth nerve and thus is unsuitable for intraoperative monitoring (30), while the former can only be recorded in small tumors in which at least a portion of the eighth nerve is accessible for correct electrode positioning. Therefore, BAEP remains an important intraoperative neuromonitoring method to preserve hearing during posterior fossa surgery.

Of the 12 included retrospective and three prospective studies, eight concluded that intraoperative BAEP aids in predicting post-operative hearing preservation (16–18, 20, 22, 24–26), corresponding with our results regarding intraoperative BAEP loss group; the four other studies held various conflicting views, focusing on low sensitivity or specificity (14, 19, 23, 28), which was reflected in our intraoperative BAEP change analyses. Specificity inconsistency may have originated from the studies' sample size, the time of each study conducted, and the varying BAEP standards. In addition, being susceptible to disruption by various operative procedures made it difficult sometimes to obtain a reliable BAEP signal. It is associated with a higher incidence of false positives. The results from the analyses herein indicate that, with a specific amount of change and until a complete loss of BAEP signals, specificity and sensitivity vary. We expect that the inclusion of studies with consistent BAEP diagnostic values would provide a clearer threshold value.

In addition to the prediction of post-operative nerve function, BAEP is important to aid the neurosurgeons' intraoperative decision on whether to continue tumor resection to protect the nerve. Four studies included were aimed at the hearing protecting function of the BAEP in assisting the surgeons in the operation at the primary level, which was also successfully proven by them (15, 21, 26, 27). Association between surgical steps and intraoperative BAEP changes was reported as another possible direction toward revealing the mechanisms of post-operative hearing loss (28, 31). Further research quantifying the signal change during different surgical steps is required to identify more sensitive intraoperative BAEP thresholds.

A more accurate diagnostic surgical tool would also allow neurosurgeons to customize their pre-surgical strategy incorporating patient preference. For example, patients who are career musicians and those with bilateral VS may choose strategies that preserve hearing. Intracochlear BAEP is used to assess the cochlear nerve integrity to help select the candidate for cochlear implantation (32). Moreover, experimental nerve regeneration therapies including electrical stimulation, electroactive surgical nanomaterials, and gene therapy might provide new hope in future (33, 34).

The limitations of the current study are as follows. Data source restrictions were a barrier to further analyses of more detailed types of changes in intraoperative BAEP. Three of the included studies used inappropriate follow-up timelines which may have influenced their hearing outcomes. Limiting the inclusion of the study to English language articles, since most of the studies were retrospective studies, may also have contributed to selection bias. The relatively small sample size is another potential source of bias. Heterogeneity was inevitable despite not identifying its significant sources in subgroup analyses, which were limited by data missing from some studies, including prognostic factors like tumor size (3, 35, 36).

## Conclusion

The loss of intraoperative BAEP has a meaningful predictive function on potential hearing loss after VS resection. The change in the wave should raise the neurosurgeons' attention in VS surgeries aimed at preserving hearing and resecting the tumor as much as possible. Further studies that focus on a more specific change in intraoperative BAEP signals with more samples should be conducted.

## Data availability statement

The original contributions presented in the study are included in the article/Supplementary material, further inquiries can be directed to the corresponding author/s.

## Author contributions

ZhW was the principal investigator. ZC designed the study protocol. FG and XY searched the databases, screened the studies, analyzed the data, and finished the manuscript. ZiW and XT revised the manuscript and polished the language. TX and GC provided professional guidance for the process of meta-analysis. All authors contributed to the article and approved the submitted version.
