# Supplementary material for: Diagnostic accuracy of intraoperative brainstem auditory evoked potential for predicting hearing loss after vestibular schwannoma surgery
Source: Front Neurol. 2022 Dec 15;13:1018324. doi: 10.3389/fneur.2022.1018324 (PMC9797509; doi:10.3389/fneur.2022.1018324)

Supplemental Digital Content 1. Methods. Search strategy

((brainstem auditory evoked potential) or (brainstem auditory evoked response) or (auditory brainstem response) or (BAEP) or (BAER) or (ABR)) and ((Intraoperative) or (during operation) or (during surgery)) and ((acoustic neuroma) or (vestibular schwannoma) or (vestibulocochlear schwannoma) or (cochlear schwannoma))

Supplemental Digital Content 2. Figure. QUADAS-2 of separate studies.


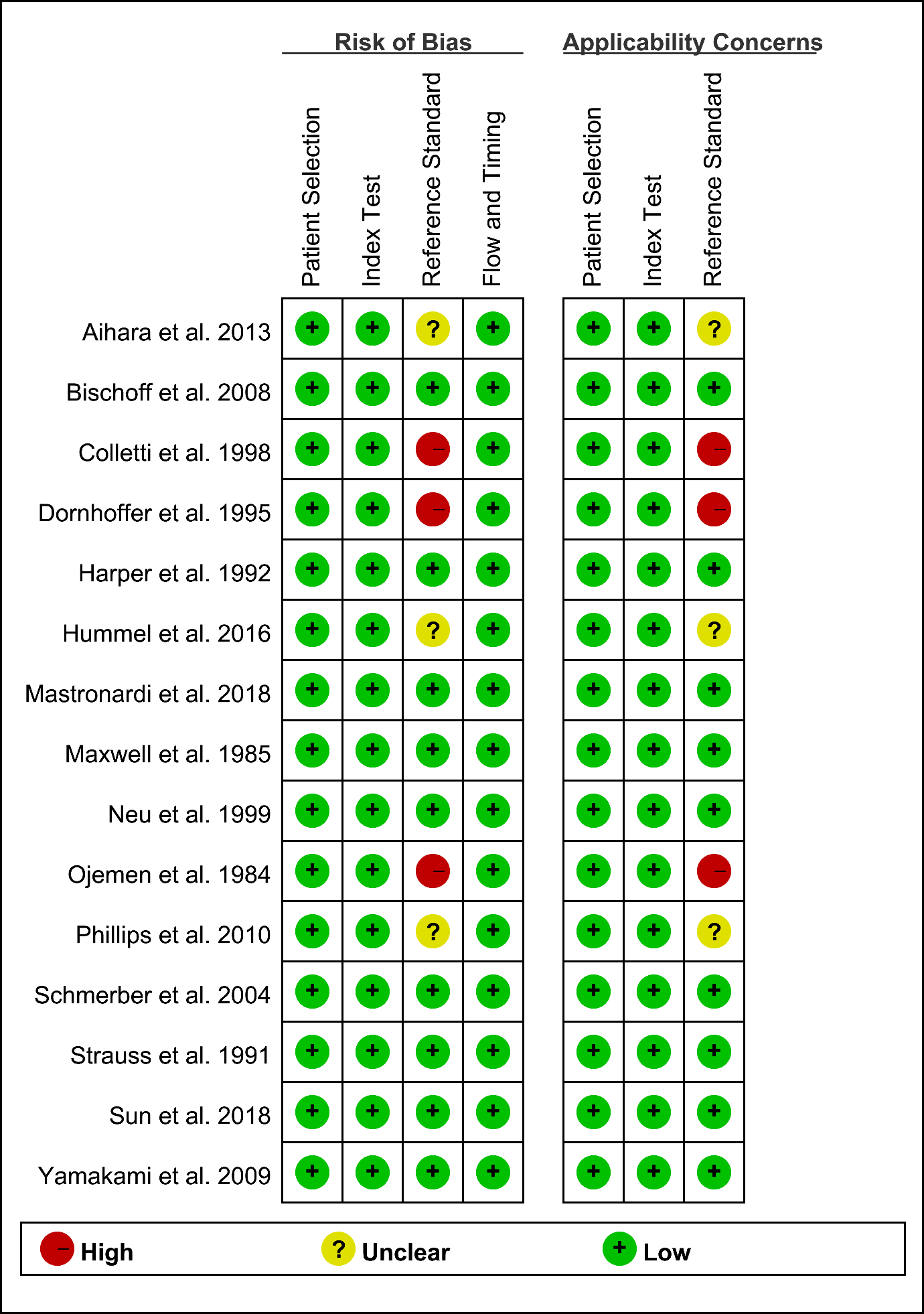


Supplemental Digital Content 3. Figure. QUADAS-2 of summary studies.


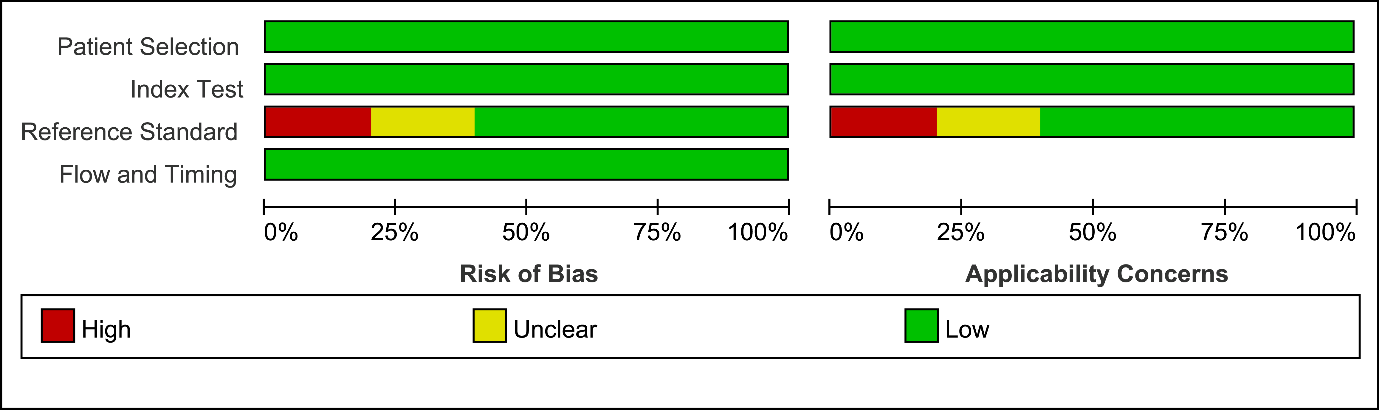


Supplemental Digital Content 4. Figure. Likelihood ratio scattergram of intraoperative BAEP changed group.


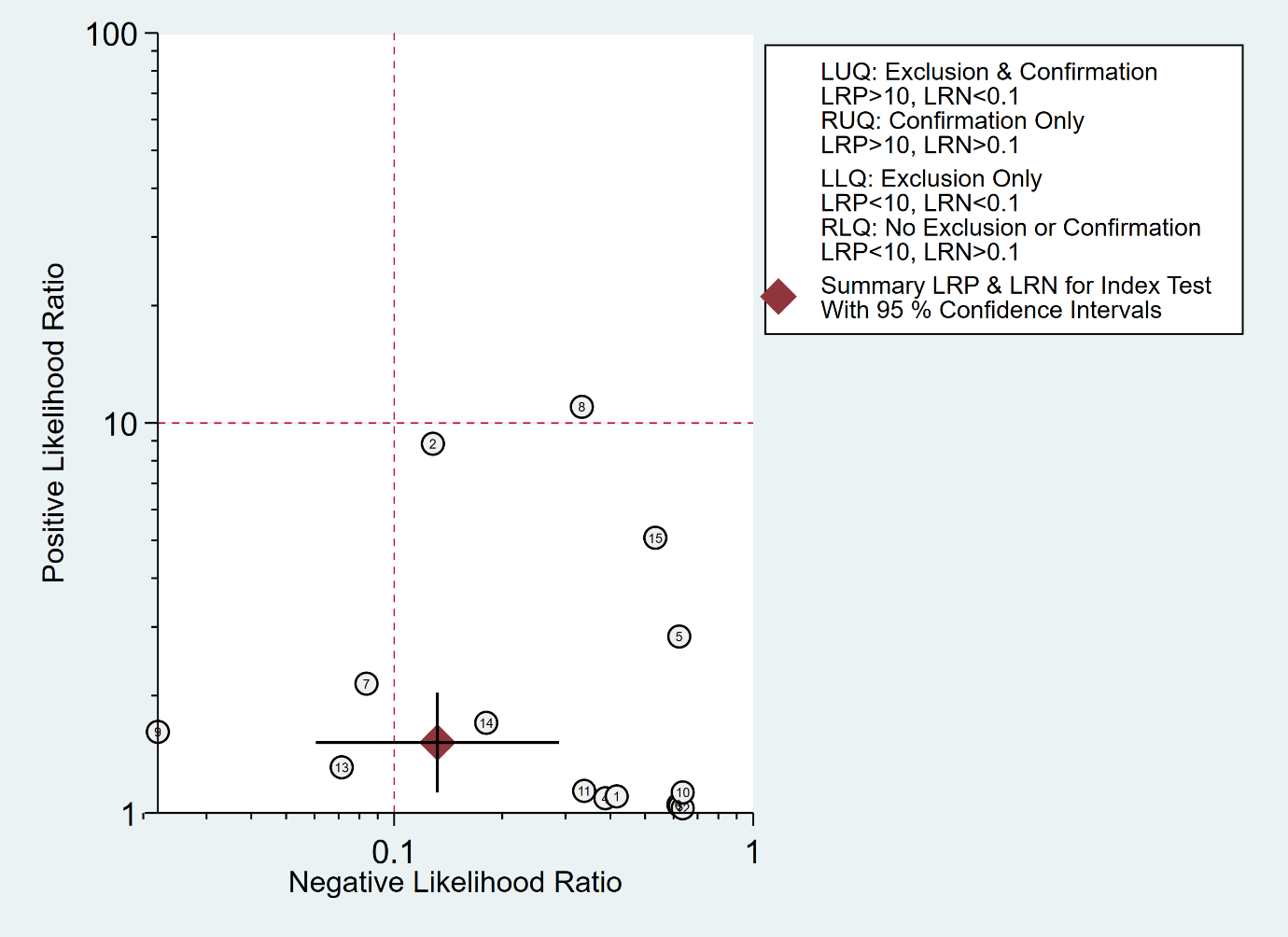


Supplemental Digital Content 5. Figure. Likelihood ratio scattergram of intraoperative BAEP lost group.


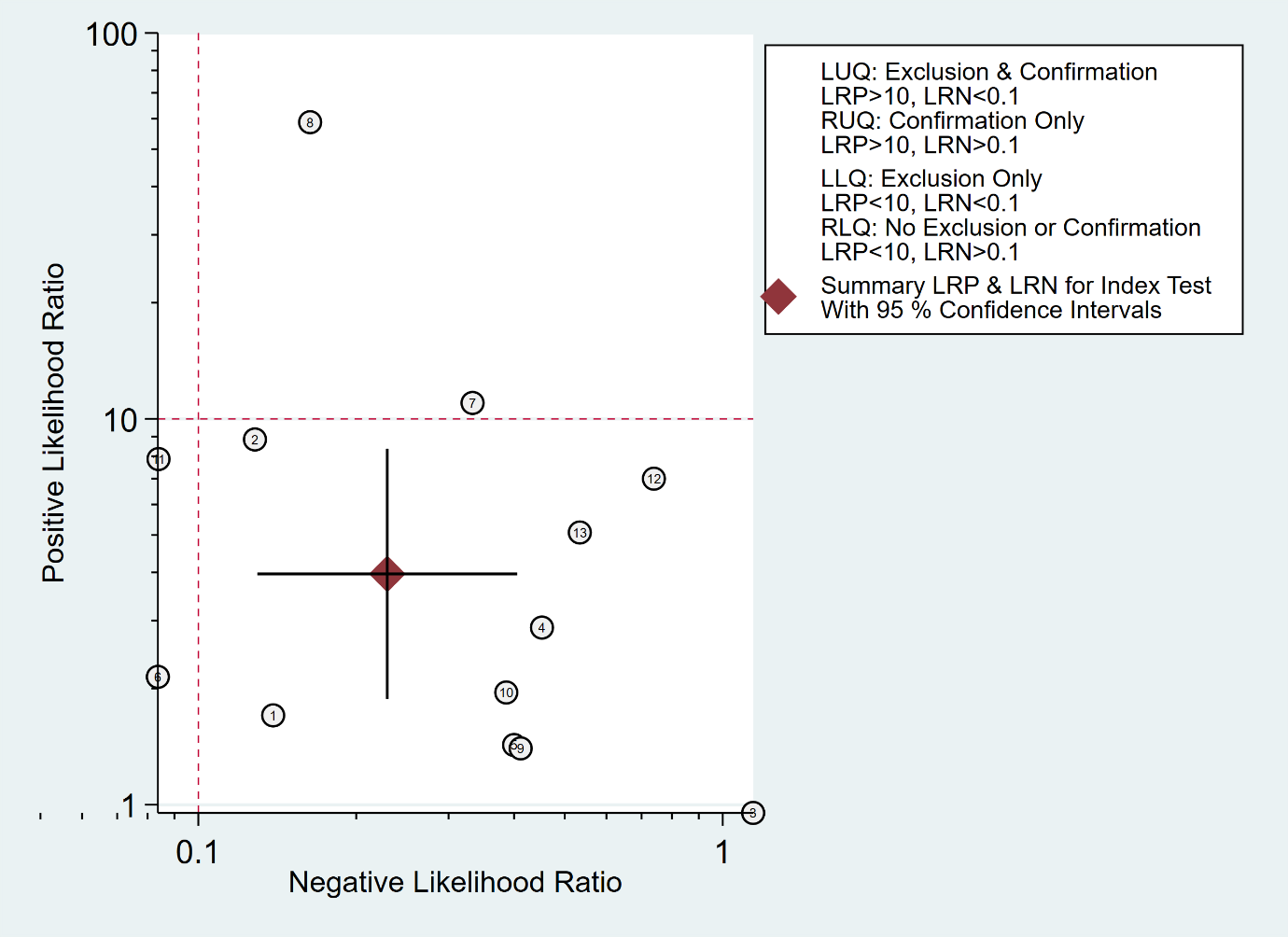


Supplemental Digital Content 6. Figure. Meta-regression & Subgroup analysis of intraoperative BAEP changed group.


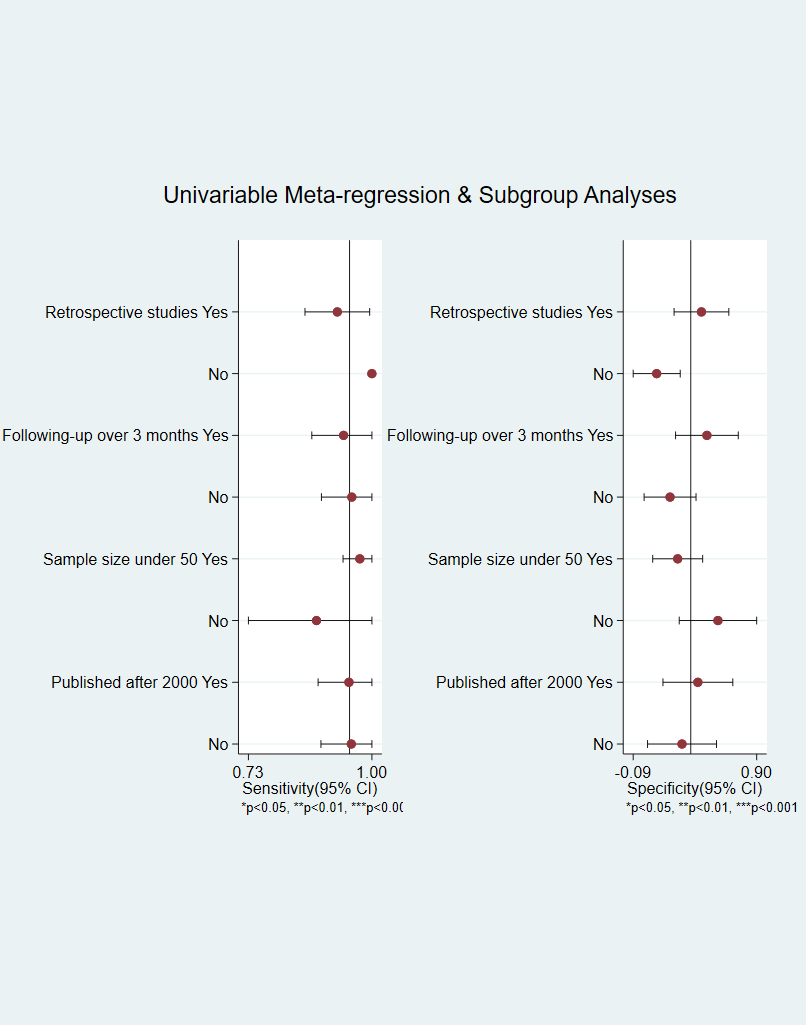


Supplemental Digital Content 7. Figure. Meta-regression & Subgroup analysis of intraoperative BAEP lost group.


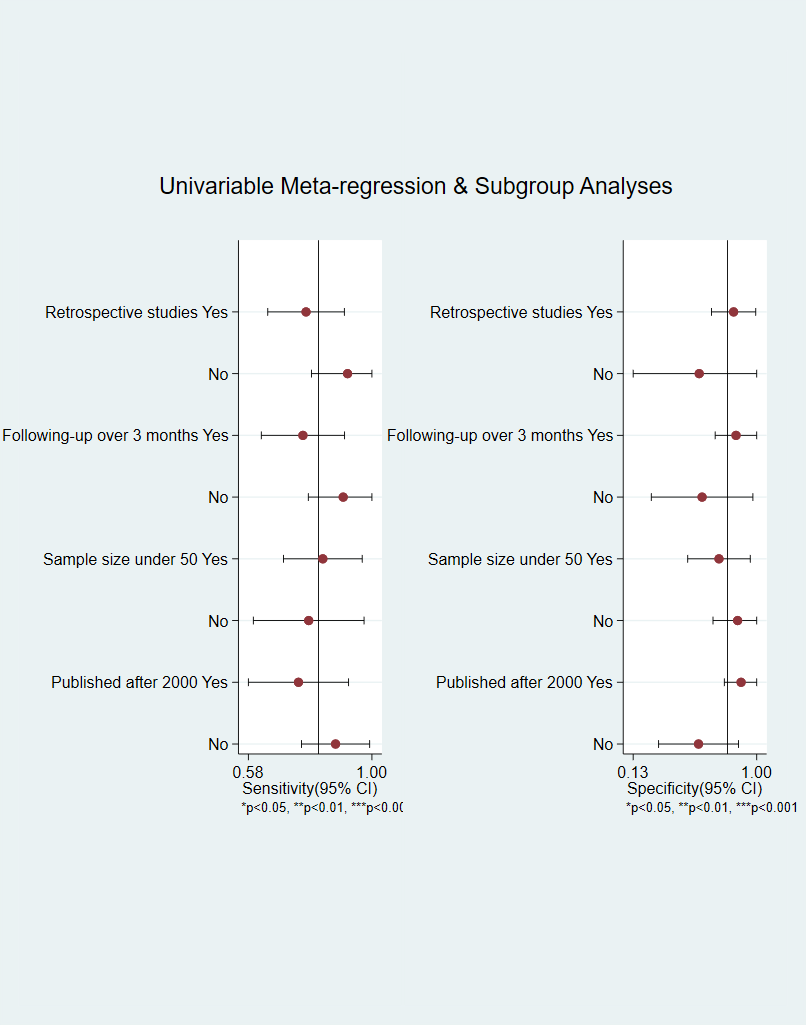


Supplemental Digital Content 8. Table. Meta-regression of intraoperative BAEP changed group.

| Parameter | Category | Sensitivity | Specificity | Chi^2^ | P-value |
| --- | --- | --- | --- | --- | --- |
| Retrospective studies | Yes | 0.92 (0.85-1.00) | 0.46 (0.24-0.68) | 5.41 | 0.07 |
|  | No | 1.00 (1.00-1.00) | 0.10 (-0.09-0.29) |  |  |
| Following-up over 3 months | Yes | 0.94 (0.87-1.00) | 0.50 (0.25-0.75) | 4.82 | 0.09 |
|  | No | 0.96 (0.89-1.00) | 0.21 (0.00-0.41) |  |  |
| Sample size under 50 | Yes | 0.97 (0.94-1.00) | 0.27 (0.06-0.48) | 2.94 | 0.23 |
|  | No | 0.88 (0.73-1.00) | 0.59 (0.28-0.90) |  |  |
| Published after 2000 | Yes | 0.95 (0.88-1.00) | 0.43 (0.15-0.71) | 0.70 | 0.70 |
|  | No | 0.95 (0.89-1.00) | 0.30 (0.03-0.58) |  |  |

Supplemental Digital Content 9. Meta-regression of intraoperative BAEP lost group.

| Parameter | Category | Sensitivity | Specificity | Chi^2^ | P-value |
| --- | --- | --- | --- | --- | --- |
| Retrospective studies | Yes | 0.78 (0.65-0.91) | 0.84 (0.68-0.99) | 2.18 | 0.34 |
|  | No | 0.92 (0.79-1.00) | 0.59 (0.13-1.00) |  |  |
| Following-up over 3 months | Yes | 0.77 (0.62-0.91) | 0.85 (0.71-1.00) | 2.62 | 0.27 |
|  | No | 0.90 (0.78-1.00) | 0.62 (0.26-0.97) |  |  |
| Sample size under 50 | Yes | 0.83 (0.90-0.97) | 0.73 (0.51-1.00) | 0.76 | 0.69 |
|  | No | 0.79 (0.60-0.97) | 0.78 (0.69-1.00) |  |  |
| Published after 2000 | Yes | 0.75 (0.58-0.92) | 0.89 (0.77-1.00) | 4.37 | 0.11 |
|  | No | 0.88 (0.76-0.99) | 0.59 (0.30-0.87) |  |  |

Supplemental Digital Content 10. Figure. Meta-regression & Subgroup analysis of intraoperative BAEP changed group excluding studies without reporting mean tumor size.


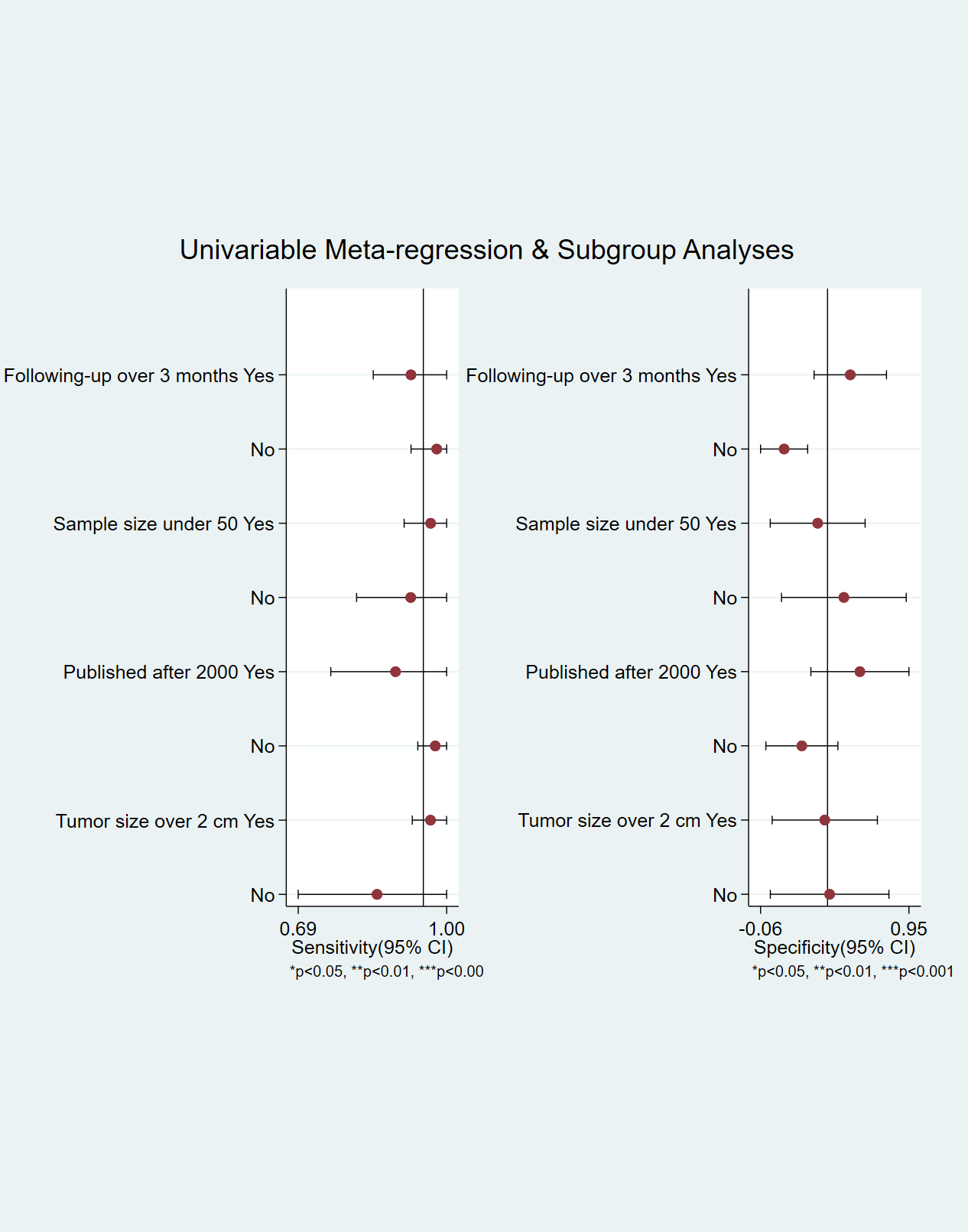


Supplemental Digital Content 11. Figure. Meta-regression & Subgroup analysis of intraoperative BAEP lost group excluding studies without reporting mean tumor size.


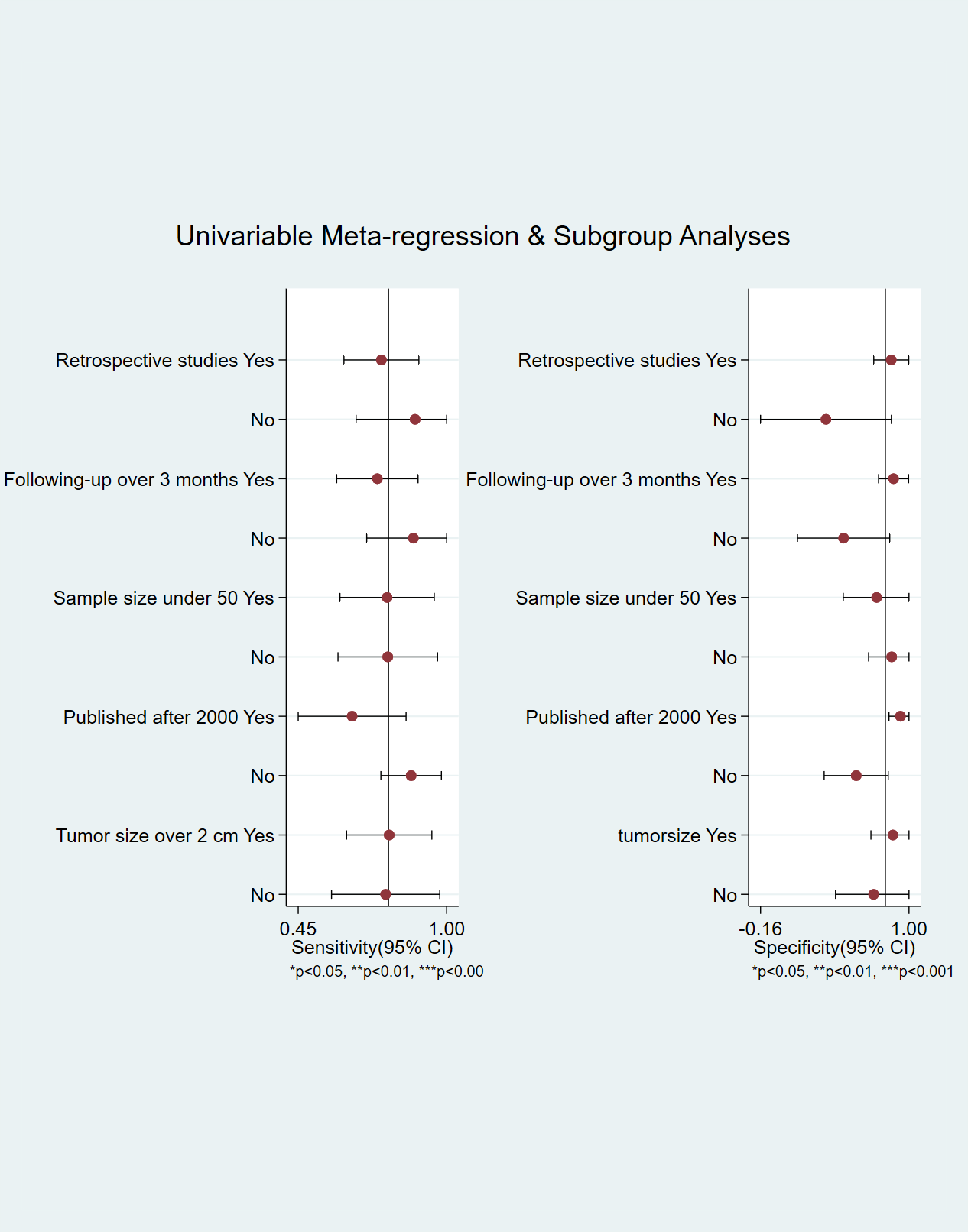

Supplement: Supplementary file 1 [file Data_Sheet_1.docx]
